# The co-expression of functional gastric proteins in dynamic gastric diseases and its clinical significance

**DOI:** 10.1186/1472-6890-13-21

**Published:** 2013-08-09

**Authors:** Qian Xu, Li-ping Sun, Ben-gang Wang, Jing-wei Liu, Ping Li, Cai-yun He, Yuan Yuan

**Affiliations:** 1 Tumor Etiology and Screening Department of Cancer Institute and General Surgery, The First Affiliated Hospital of China Medical University, North Nanjing Street 155#, Heping District, Shenyang 110001, People’s Republic of China; 2 Key Laboratory of Cancer Etiology and Prevention, (China Medical University), Liaoning Provincial Education Department, Shenyang 110001, People’s Republic of China; 3 Department 1 of General Surgery in General Surgery Institute, The First Affiliated Hospital of China Medical University, Shenyang 110001, China

**Keywords:** Pepsinogen C(PGC), Mucin1(MUC1), Mucin2 (MUC2), Immunohistochemistry, Gastric cancer, Functional proteins

## Abstract

**Background:**

Pepsinogen C (PGC) and mucin1 (MUC1) are important physiologically functional gastric proteins; Mucin2 (MUC2) is an “ectopic” functional protein in intestinal metaplasia of gastric mucosa. We analyzed the co-expression of the above-mentioned three proteins in dynamic gastric diseases {superficial gastritis (SG)-atrophic gastritis (AG)--gastric cancer (GC)} as well as different histological types of gastric cancer in order to find molecular phenotypes of gastric cancer and precancerous disease and further explore the potential co-function of PGC, MUC1 and MUC2 in the occurrence and development of gastric cancer.

**Methods:**

The SG-AG-GC sequence was 57-57-70 cases in this case–control study, respectively. Different histological types of GC were 28 cases of highly and moderately differentiated aden ocarcinoma (HMDA)、30 of poorly differentiated adenocarcinoma (PDA) and 12 of mucinous adenocarcinoma (MA) or signet ring cell carcinoma (SRCC). PGC, MUC1 and MUC2 expression in situ were detected in all 184 cases using immunohistochemistry.

**Results:**

Both PGC and MUC1 had a significantly decreased expression in GC than in SG and AG (*P* < 0.0001 and *P* < 0.01, respectively); While MUC2 had a significant increased expression in AG than in SG and GC (*P* < 0.0001). Seven phenotypes of PGC, MUC1 and MUC2 co-expression were found in which PGC+/MUC1+/MUC2- phenotype took 94.7%(54/57) in SG group; PGC+/MUC1+/MUC2+ and PGC-/MUC1+/MUC2+ phenotype took 43.9% (25/57) and 52.6% (30/57) in AG; the phenotypes in GC group appeared variable; extraordinarily, PGC-/MUC1-/MUC2+ phenotype took 100% (6/6) in MA or SRCC group and had a statistical significance compared with others (*P* < 0.05).

**Conclusions:**

Phenotypes of PGC, MUC1 and MUC2 co-expression in dynamic gastric diseases are variable. In SG group it always showed PGC+/MUC1+/MUC2- phenotype and AG group showed two phenotypes (PGC+/MUC1+/MUC2+ and PGC-/MUC1+/MUC2+); the phenotypes in GC group appeared variable but the phenotype of PGC-/MUC1-/MUC2+ may be a predictive biomarker for diagnosing MA or SRCC, or distinguishing histological MA or SRCC from tubular adenocarcinoma accompanied by mucinous secretion or signet ring cell scattered distribution.

## Background

Normal function of stomach needs two kinds of materials which mainly exist in normal gastric juice. They are protein components and small molecular materials participating in regulation. Among them, only pepsinogen and mucin belong to protein components in the gastric juice which are both important physiologically functional gastric proteins. Pepsinogens were divided into pepsinogen A (PGA) and pepsinogen C (PGC), and the latter is a precursor of human pepsin C which is a digestive enzyme [1]. The appearance of PGC is a signal of gradually maturing of digestive function [2]. 1% of human PGC from the stomach was secreted into peripheral blood and the ratio of PGA and PGC detected in the serum was a biomarker for atrophic gastritis (AG) or gastric cancer (GC) [3-5]. Mucin (MUC) is a kind of glycoprotein family secreted by mucosal epithelium possessing functions of protection, lubrication and hydration for epithelial lumens, and 20 kinds of mucins had been identified from MUC1 to MUC21 until now [6]. MUC1 is a highly polymorphic membrane-associated mucin containing core peptide chain and glycoprotein side chain. MUC1 possesses a protective capacity which participates in composing the barrier of “mucus-bicarbonate” , and also functions in a cell signaling capacity [7,8]. Its overexpression or aberrant intracellular localization is found in cancerous cells and is associated with carcinomas [9-12].

Except the above-mentioned physiologically functional gastric proteins, an “ectopic” functional protein, MUC2 could be expressed when the gastric mucosa occurred intestinal metaplasia. MUC2 is also a highly polymorphic mucin containing core peptide chain and glycoprotein side chain [13,14]. In the normal physiological circumstances, the MUC2 protein expresses in the intestinal mucosa and is absent in the normal gastric mucosa. However, MUC2 could be “ectopic” expressed at gastric mucosa under pathogenic effect of external factors. Whether it participates in the response of the host to these external pathogenic factors such as the inflammation [15] or *H. pylori*[16,17] remains controversial.

As physiologically functional gastric proteins and “ectopic” expressed protein, the association of PGC, MUC1 and MUC2 solely and gastric diseases had been reported in the past [18-22]. Our team had also demonstrated in previous study that PGC expression had a close relationship with the degree of malignancy of gastric mucosa [23], and we also found MUC1 protein had a significant underexpression in GC compared with non-cancer subjects [24]. Additionally, MUC2 expression had a close relationship with atrophic gastritis [25]. However, analysis of these proteins in a same group of cases representing different stages of cancer progression had never been reported until now, which may provided some new insight in the possible function of these proteins, and also in the possible molecular phenotypes of different gastric diseases.

In this case–control study, we investigated the co-expression of PGC, MUC1 and MUC2 in situ in the same group of cases in the SG-AG-GC sequence, as well as in different histological types of GC in order to find molecular phenotypes of gastric cancer and precancerous disease and further explore the potential co-function of PGC, MUC1 and MUC2 in the occurrence and development of gastric cancer.

## Methods

### Patients

This research project was approved by the Ethical Committee of the China Medical University, and all the gastric tissue specimens of 184 patients were collected from patients with letters of consent and questionnaire of medical history who participated in a health check program by gastroscopy for gastric cancer screening or in hospitals located in Zhuanghe and Shenyang of Liaoning Province in China between 2002 and 2005. Patients with a history of other malignant neoplasms or other gastric benign diseases including gastric erosion, peptic ulcer diseases, gastric polyp, and adenomas were excluded. The biopsy specimens from the gastroscopies were paraffin-embedded and stained by hematoxylin and eosin (HE) staining method; the eligibility criterion for all the cases is histological diagnosis. The biopsy specimens were collected from 70 GC patients with an average age of 59.13 ± 10.50 years ranging from 34 to 80 years old. 57 patients with superficial gastritis (SG) and 57 patients with atrophic gastritis (AG) had a similar average age of 57.18 ± 11.74 and 57.88 ± 10.62 years respectively, ranging from 34 to 79 years old. The GC and SG, AG groups had no statistical difference in terms of gender and age composition (*P* = 0.812 and *P* = 0.593, respectively, Table 1). Furthermore, we classified samples of 70 GC patients according to WHO classification (28 HMDA, 30 PDA and 12 MA or SRCC). The HMDA, PDA and MA or SRCC subgroups showed no statistical difference in gender and age composition (*P* = 0.310 and *P* = 0.141, respectively, Table 1).

**Table 1:** The basic messages of the objects

**Variability**	**SG**	**AG**	**GC**	**GC analyzed for PGC, MUC1 and MUC2 (n = 70)**		
				**HMDA**	**PDA**	**MA or SRCC**
	n = 57	n = 57	n = 70	n = 28	n = 30	n = 12
Sex						
Male	36	38	48	20	22	6
Female	21	19	22	8	8	6
	*P* = 0.812			*P* = 0.310		
Age						
Average	57.88 ± 10.62	57.18 ± 11.74	59.13 ± 10.50	61.54 ± 9.62	58.77 ± 11.05	54.42 ± 10.17
Range	42-79	34-79	34-80	42-80	35-80	34-71
	*P* = 0.593			*P* = 0.141		

### Immunohistochemical staining for detection of PGC, MUC1 and MUC2 protein expression in situ

Immunohistochemical analysis was performed in 5-μm-thick sections from sequentially sliced samples of formalin-fixed and paraffin-embedded specimens according to the method described by Byrd [24,26] with slight modification. Briefly, tissue sections were deparaffinized and rehydrated. Endogenous peroxidase was blocked using 3% hydrogen peroxide in methanol for 10 min and then the sections were washed with phosphate buffered saline (PBS), pH 7.4. The sections were incubated with non-immunized horse serum for 20 min at room temperature and washed before being incubated with a specific antibody overnight at 4°C. Then the sections were washed and incubated with biotinylated secondary antibodies (goat anti-mouse antibody, Maixin Inc., Fujian, China) and streptavidin-biotin peroxidase. After three washes with PBS, the sections were visualized with 3,3’- diaminobenzidine tetra-hydrochloride and counterstained with haematoxylin. Primary antibodies were replaced with PBS buffer as a negative control. The specific mouse anti-human antibodies were purchased from Neomarkers Inc. Fremont, USA (Human Milk Fat Global-1, HMFG-1, against MUC1, 1:200 dilutions) [27,28] and Maixin Inc. Funjian, China (against MUC2, clone No. M53, 1:100 dilutions). And mouse anti-human antibody against PGC (clone No. 2D5, 1:500 dilutions) was presented by Clinical Laboratory Institute of Japanese, kindly. Immunohistochemical results were judged by HSCORE (histological score) [29]. The HSCORE was calculated using two indices of proportion (Pί) and intensity (ί). The proportion (Pί) was estimated after taking into account the percentage of positive cells. The intensity (ί) was judged as 0 (no staining), 1+ (light brown staining), 2+ (brown staining), or 3+ (heavy brown staining). The HSCORE was derived by summing the proportion of cells staining intensity multiplied by the intensity of staining.

HSCORE=∑Pί×ί

Where ί = 0, 1, 2, 3 and Pί varies from 0.0 to 1.0, the HSCOREs ranged from a minimum of zero in cases with no staining to a maximum of 3.0 in cases in which all the cells were stained with maximal intensity. We judged HSCORE > 0.0 as positive while HSCORE = 0.0 as negative. The HSCORE was determined by two independent observers.

### Statistics

Non parametric test and Fisher’s exact probabilities were used to determinate the difference of PGC, MUC1, MUC2 expression in the SG-AG-GC sequence and in different histological types of GC. χ^2^ test and Fisher’s exact probabilities were used to determinate the difference of the co-expression of the three proteins. The rank-sum test and Spearman’s rank correlation analysis were used to analyze the correlations in different groups. All statistical analysis was performed using SPSS (16.0) statistical software program (SPSS, Chicago, USA). All the statistical test was two-side probability test and *P*<0.05 was considered as statistically significant.

## Results

### The expression of PGC, MUC1, MUC2 in GC and precancerous diseases groups

**Figure 1:**
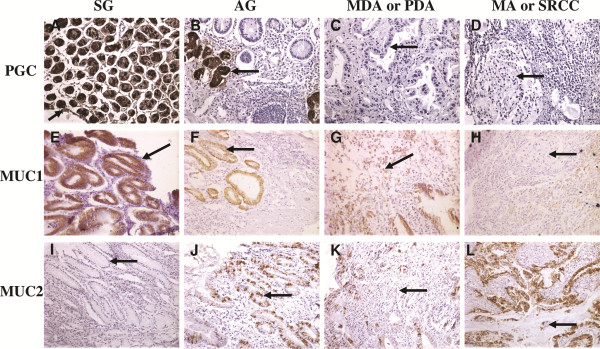
**The expression of PGC, MUC1, MUC2 proteins in different gastric mucosa tissues (× 200). A**. strong positive expression of PGC in the gastric mucosa of superficial gastritis (SG) group; **B**. positive expression of PGC in atrophic gastritis (AG) group, a few negative expression; **C**. negative expression of PGC in moderately differentiated adenocarcinoma (MDA) group; **D**. negative expression of PGC in signet ring cell carcinoma (SRCC) group. **E**. strong positive expression of MUC1 in SG group; **F**. positive expression of MUC1 in AG group; **G**. positive expression of MUC1 in poorly differentiated adenocarcinoma (PDA) group; **H**. negative expression of MUC1 in SRCC group; **I**. negative expression of MUC2 in SG group; **J**. strong positive expression of MUC2 in AG group; **K**. positive expression of MUC2 in PDA group; **L**. strong positive expression of MUC2 in mucinous adenocarcinoma group. The arrow all means the cells of pathological changes.

PGC expression was found in the cytoplasm of gastric mucosal cells (Figure 1A). MUC1 expression was found in the cytoplasm and/or membrane of gastric mucosal cells (Figure 1E). MUC2 protein was colored in the cytoplasm of goblet cells (Figure 1J).

The expression frequency of the three proteins in different gastric disease groups was examined by immunohistochemical analysis. PGC and MUC1 proteins expression in the GC group was significantly lower than that in the SG and AG groups (*P* < 0.05). MUC2 protein expression in the GC group was significantly lower than that in the AG group (*P* < 0.001), but the expression of SG group was lower than that in GC group (*P* < 0.001). The correlation of PGC, MUC1 and MUC2 protein expression with SG-AG-GC sequence was analyzed respectively. We found there were correlations between these three proteins and the disease sequence (*P* < 0.01); PGC and MUC1 showed negative correlations with the SG-AG-GC sequence (correlation coefficients were r = −0.770, r = −0.210, respectively; *P* < 0.001, *P* = 0.004, respectively ). MUC2 indicated positive correlation with the SG-AG-GC sequence (correlation coefficient was r = 0.260, *P* < 0.001). Furthermore, according to WHO histological classification, the GC group was divided into high and moderated adenocarcinoma (HMDA), poorly differentiated adenocarcinoma (PDA), mucinous adenocarcinoma or signet ring cell carcinoma group (MA or SRCC) groups. We found that MUC1 protein in MA or SRCC group was significantly lower than that in the HMDA and PDA groups (*P* < 0.001, Table 2), and MUC2 in MA or SRCC group was significantly higher than that in the PDA groups (*P* = 0.013, Table 2), but PGC protein had no significant difference in the GC group.

**Table 2:** Frequency of PGC, MUC1 and MUC2 protein expression in gastric diseases

**Staining score**	**SG**	**AG**	**GC**	**GC(n = 70)**		
				**HMDA**	**PDA**	**MA or SRCC**
	**n = 57**	**n = 57**	**n = 70**	**n = 28**	**n = 30**	**n = 12**
PGC						
2.0-3.0	19(33.3)	2(3.5)	0(0.0)	0(0.0)	0(0.0)	0(0.0)
1.0-1.9	29(50.9)	14(24.6)	0(0.0)	0(0.0)	0(0.0)	0(0.0)
0.1-0.9	9(15.8)	13(22.8)	0(0.0)	0(0.0)	0(0.0)	0(0.0)
0	0(0.0)	28(49.1)	70(100.0)	28(100.0)	30(100.0)	12(100.0)
*P*_*1*_	<0.0001	<0.0001	-			
*P*_*2*_				1.000	1.000	-
MUC1						
2.0-3.0	24(42.1)	29(50.9)	21(30.4)	12(42.9)	9(31.0)	0(0.0)
1.0-1.9	19(33.3)	19(33.3)	15(21.7)	8(28.6)	6(20.7)	1(8.3)
0.1-0.9	13(22.8)	9(15.8)	23(33.3)	7(25.0)	12(41.4)	4(33.0)
0	1(1.8)	0(0.0)	10(14.5)	1(3.6)	2(6.9)	7(58.3)
*P*_*3*_	0.012	0.0003	-			
*P*_*4*_				<0.0001	0.0004	-
MUC2						
2.0-3.0	2(3.5)	46(80.7)	12(17.1)	6(21.4)	2(6.7)	4(33.3)
1.0-1.9	0(0.0)	6(10.5)	8(11.4)	3(10.7)	2(6.7)	3(25.0)
0.1-0.9	0(0.0)	3(5.3)	19(27.1)	9(32.1)	8(26.7)	2(16.7)
0	55(96.5)	2(3.5)	31(44.3)	10(35.7)	18(60.0)	3(25.0)
*P*_*5*_	<0.0001	<0.0001	-			
*P*_*6*_				0.273	0.013	-
*P*_*7*_	<0.0001	<0.0001		-		
*P*_*8*_	<0.0001	<0.0001			-	
*P*_*9*_	<0.0001	0.001				-

Note: Statistical analysis used nonparametric tests and Fisher’s exact tests. *P*_*1*_*, P*_*3*_*, P*_*5*_ means the significance compared with GC group, respectively. *P*_*2*_*, P*_*4*_*, P*_*6*_ means the significance compared with MA or SRCC group, respectively. *P*_*7*_ means the significance compared with HMDA group; *P*_*8*_ means the significance compared with PDA group; *P*_*9*_ means the significance compared with MA or SRCC group.

### The co-expression phenotype of PGC, MUC1, MUC2 in GC and precancerous disease groups

**Figure 2:**
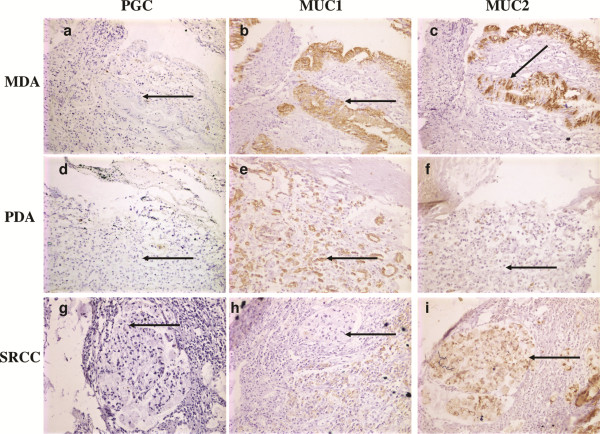
**The phenotype of PGC/MUC1/MUC2 co-expression in the same pathological changes (× 200). A**. The negative expression of PGC in moderately differentiated adenocarcinoma (MDA) group. **B**. The positive expression of MUC1 in MDA group. **C**. The positive expression of MUC2 in MDA group. **D**. The negative expression of PGC in poorly differentiated adenocarcinoma (PDA) group. **E**. The positive expression of MUC1 in PDA group. **F**. The negative expression of MUC2 in PDA group. **G**. The negative expression of PGC in signet ring cell carcinoma (SRCC) group. **H**. The negative expression of MUC1 in SRCC group. **I**. The positive expression of MUC2 in SRCC group. Every three figures in a horizontal composition were all from the same individual. The arrow all means the cells of pathological changes.

The co-expression characteristics of PGC, MUC1, MUC2 proteins were analyzed. We found the phenotype of PGC+/MUC1+/MUC2- accounted for 94.7% (54/57) in SG group; PGC+/MUC1+/MUC2+ and PGC-/MUC1+/MUC2+ phenotype accounted for 43.9% (25/57) and 52.6% (30/57) in AG group, respectively. The phenotypes in GC group appeared variable, among them, all cases were PGC- phenotype, in which most were individuals with phenotype of PGC-/MUC1+/ MUC2+ or PGC-/MUC1+/MUC2- (33 or 26 cases, accounted for 47.1% and 37.1% respectively, as shown in Figure 2 A-C or D-F). Furthermore, the GC cases were classified according to histological types, we found that 100% (6/6) in the MA or SRCC group were the phenotype of PGC-/MUC1-/MUC2+ (Figure 2 G-I), other phenotypes group in the MA or SRCC group were significantly lower compared with that of PGC-/MUC1-/MUC2+ phenotype group (*P* < 0.05, Table 3).

**Table 3:** Different phenotype of the concordance of PGC, MUC1 and MUC2 protein expression in gastric diseases

**Factors**			**n**	**Different gastric diseases**			**Different histological type of GC**			
**PGC**	**MUC1**	**MUC2**		**SG(n = 57)**	**AG(n = 57)**	**GC(n = 70)**	**HMDA(n = 28)**	**PDA(n = 30)**	**MA or SRCC(n = 12)**	P^*^
-	-	-	5	-	-	5 (100.0)	1 (20.0)	3 (60.0)	1 (20.0)	0.015
-	-	+	6	-	-	6 (100.0)	-	-	6 (100.0)	-
-	+	-	27	-	1 (3.7)	26 (96.3)	9 (34.6)	15 (57.7)	2 (7.7)	<0.0001
-	+	+	63	-	30 (47.6)	33 (52.4)	18 (54.8)	12 (38.7)	3 (6.5)	<0.0001
+	-	-	1	1 (100.0)	-	-	-	-	-	-
+	-	+	0	-	-	-	-	-	-	-
+	+	-	55	54 (98.2)	1 (1.8)		-	-	-	-
+	+	+	27	2 (7.4)	25 (92.6)		-	-	-	-

Note:^*^P value meant other group compared with PGC-/MUC1-/MUC2+ phenotype group and used Fisher’s exact probability.

The Spearman correlation analysis showed that every two of PGC, MUC1 and MUC2 had correlation (*P* < 0.05). There was a positive correlation between PGC and MUC1 (*P* = 0.006, r = 0.200), as well as MUC1 and MUC2 (*P* = 0.177, r = 0.016), while PGC and MUC2 had a negative correlation but the correlation coefficient was the largest (*P* < 0.001, r = −0.313).

## Discussion

Pepsinogen C (PGC) and mucin1 (MUC1) are important physiologically functional gastric proteins; Mucin2 (MUC2) is an “ectopic” functional protein in intestinal metaplasia of gastric mucosa. We analyzed the co-expression of the these three proteins in a dynamic gastric disease sequence as well as different histological types of gastric cancer in order to explore the co-expression-based molecular phenotypes of gastric cancer as well as its precancerous disease and correlation between the co-expression pattern and different gastric diseases.

As we know, PGC, MUC1, MUC2, the three proteins had solely important diagnostic role for the gastric disease. But the co-expression as well as their molecular phenotype had not been reported until now, while in distinction between different gastric diseases. In fact, the co-expression of combined proteins suggesting molecular phenotype like MUC2 and CD10 had been reported previously. Wakatsuki, K et al. and Hasuo, T et al. divided GC into the gastric phenotype (G-type) and intestinal phenotype (I-type) according to MUC5AC, MUC6, MUC2, and CD10 [30,31], while other scholars divided G-type and I-type according to MUC2 with other proteins [20,32]. But the significance of these studies was all based on the consideration that different phenotypes had different patterns progressing to GC. We aimed to investigate the co-expression of PGC, MUC1 and MUC2 which might also give a clue for the understanding of the progression of gastric diseases.

There were seven types of the co-expression for the studied three proteins in this study. In SG group it always showed the phenotype of PGC+/MUC1+/MUC2- which means PGC and MUC1 were both positive and MUC2 was negative. We also analyzed the correlations between the three proteins and the SG-AG-GC sequence and found that PGC and MUC1 both had negative correlation and only MUC2 had positive correlation with the disease sequence, which means PGC and MUC1 had similar distribution tendency while PGC had a larger correlation coefficient than MUC1 (r = −0.770 and r = −0.210, respectively). In AG group the co-expression of these three proteins showed two phenotypes (PGC+/MUC1+/MUC2+ and PGC-/MUC1+/MUC2+) and the main difference between them was whether PGC protein was expressed. PGC is a signal of gradually maturing of digestive function, a differentiation product of the digestive enzyme pepsin C, and it was reported that PGC gradually decreased in the SG–AG-GC sequence [23]. In our study, PGC was nearly 50% positive and 50% negative in the AG group. In the dynamic change of SG to AG, part of samples appeared PGC negative while part of samples remained positive. Among the 50% positive samples, there still were 22.8% of the cases (13/57, Table 2) which were weak positive (scored 0.1-0.9). Why part of samples appeared negative while some still remained positive? There may be two hypotheses to explain: first, it may be associated with the degree of glandular atrophy; second, the genetic variability of human PGC between individuals may contribute to the different expression of PGC in AG group. Although the latter was just a hypothesis, other protein like hypoxic marker carbonic anhydrase (CA) IX had been reported that genetic methylation status was contribute to the different expression of CA IX in GC group [33]. These hypotheses need to be investigated in the future. In GC group the co-expression of these three proteins appeared variable, but we found an interesting phenomenon. When analyzing different histological GC groups, we found the phenotype of PGC-/MUC1-/MUC2+ all distributed in the group of mucinous adenocarcinoma or signet ring cell carcinoma (MA or SRCC, 100%, 6/6). In the clinical pathological diagnosis, the histological mucinous adenocarcinoma and signet ring cell carcinoma were always hard to distinguish with tubular adenocarcinoma accompanied by mucinous secretion or signet ring cell scattered distribution. In our study, the phenotype of PGC-/MUC1-/MUC2+ all distributed in the group of MA or SRCC rather than other groups, which suggested that the histological worse differentiation MA or SRCC lost PGC and MUC1 which are biomarkers of mature differentiation. We found MUC1 positive in tubular adenocarcinoma. Even though it accompanied mucinous secretion or signet ring cell scattered distribution, it suggested a better differentiation so that biomarkers of mature differentiation like MUC1 and PGC could appear. The phenotype of PGC-/MUC1-/MUC2+ may be a predictive biomarker for diagnosing MA or SRCC or distinguishing from tubular adenocarcinoma accompanied by mucinous secretion or signet ring cell scattered distribution. Choi and his colleagues found mucinous adenocarcinoma always showed MUC1- and MUC2+ in a study of 133 MA cases [34], which was consistent with the result of our study. Could MUC2 solely identify mucinous adenocarcinoma? Probably no because it was not specific since part of the MUC2+ phenotype of GC belongs to tubular adenocarcinoma. After adding MUC1 which was a biomarker of mature differentiation to limit, the identification of MA or SRCC turned out to be more sensitive.

It is fully aware that our study had some limitations. First, only gastroscopic biopsy specimens were adopted and limited clinical data (only age and gender) were available. Second, the sample of the cases was relatively small especially GC group and its subgroup analysis. Future larger sample study was required to validate our result. Third, precancerous diseases only included AG group while other types of precancerous diseases such as adenomas were not assessed because the sample size of other precancerous diseases like adenomas was too small to be a group. Fourth, the current study only discussed two mucins (i.e. MUC1 and MUC2) and one pepsinogen PGC without inclusion of other mucins like MUC4 which has been reported increased expression in different types of gastric cancer like adenocarcinoma and SRCC [35] or other gastric functional proteins like pepsinogen A [36] and trefoil factors family [37-40]. Further investigation including stomach-related proteins could give a profile of the progression and also biomarker of gastric diseases.

## Conclusions

In conclusion, we investigated the co-expression of PGC, MUC1 and MUC2 in situ of the SG-AG-GC sequence, as well as in different histological types of GC. We found that SG showed PGC+/MUC1+/MUC2- phenotype and AG showed PGC+/MUC1+/MUC2+ and PGC-/MUC1+/MUC2+ phenotypes. The phenotype of PGC-/MUC1-/MUC2+ may be a predictive biomarker for diagnosing MA or SRCC, or distinguishing histological MA or SRCC from tubular adenocarcinoma accompanied by mucinous secretion or signet ring cell scattered distribution. The association between co-function of PGC, MUC1 and MUC2 and the occurrence and development of gastric cancer and precancerous disease needs to be clarified in the future.

## Abbreviations

SG: Superficial Gastritis; AG: Atrophic Gastritis; GC: Gastric Cancer; HMDA: High and Moderately Differentiated Adenocarcinoma; PDA: Poorly Differentiated Adenocarcinoma; MA: Mucinous Adenocarcinoma; SRCC: Signet Ring Cell Carcinoma.

## Competing interests

All authors read and approved the final manuscript, and do not have a commercial or other association that might pose a conflict of interest.

## Authors’ contributions

YY conceived and designed this study and revised the manuscript. QX was responsible for the whole experiment and involved in writing the paper. LP-S and PL were responsible for part of the experiment. BG-W collected the samples partly. CY-H performed data interpretation. JW-L participated in the revision of the manuscript. All authors read and approved the final manuscript.

## Pre-publication history

The pre-publication history for this paper can be accessed here:

http://www.biomedcentral.com/1472-6890/13/21/prepub
